# Correlation between Differential Fast Scanning Calorimetry and Additive Manufacturing Results of Aluminium Alloys

**DOI:** 10.3390/ma15207195

**Published:** 2022-10-15

**Authors:** Olaf Kessler, Evgeny Zhuravlev, Sigurd Wenner, Steffen Heiland, Mirko Schaper

**Affiliations:** 1 Chair of Materials Science, Rostock University, 18051 Rostock, Germany; 2 Competence Centre °CALOR, Department Life, Light and Matter, Rostock University, 18051 Rostock, Germany; 3 SINTEF Stiftelsen for Industriell og Teknisk Forskning, 7465 Trondheim, Norway; 4 Chair of Materials Science, Paderborn University, 33098 Paderborn, Germany

**Keywords:** PBF-LB/M, aluminium alloys, hot cracking, rapid solidification, differential fast scanning calorimetry, undercooling, grain size, crack density

## Abstract

High-strength aluminium alloy powders modified with different nanoparticles by ball milling (7075/TiC, 2024/CaB6, 6061/YSZ) have been investigated in-situ during rapid solidification by differential fast scanning calorimetry (DFSC). Solidification undercooling has been evaluated and was found to decrease with an increasing number of nanoparticles, as the particles act as nuclei for solidification. Lower solidification undercooling of individual powder particles correlates with less hot cracking and smaller grains in the material produced by powder bed fusion of metals by a laser beam (PBF-LB/M). Quantitatively, solidification undercooling less than about 10–15 K correlates with almost crack-free PBF-LB/M components and grain sizes less than about 3 µm. This correlation shall be used for future purposeful powder material design on small quantities before performing extensive PBF-LB/M studies.

## 1. Introduction

Powder bed fusion of metals by a laser beam (PBF-LB/M) is a very attractive production process due to its high flexibility and a high degree of geometrical freedom. Local microstructures and properties of PBF-LB/M components are strongly influenced by process inherent complex temperature/time profiles with multiple rapid melting/solidification cycles and multiple rapid heating/cooling cycles. Considering lightweight applications, PBF-LB/M is very promising for high-strength aluminium alloys. Unfortunately, PBF-LB/M of high-strength aluminium alloys is strongly affected by hot cracking. When cooling along the relatively large solidification intervals (e.g., about 80 K in equilibrium for alloy 7021 [1]), the remaining melt is encapsulated between the growing dendrites. During the final solidification, these melt areas shrink. If this shrinkage cannot be accommodated by deformation of the surrounding solid network, hot cracks can occur [2,3,4]. One successful approach to overcome hot cracking during PBF-LB/M of high-strength aluminium alloys is the addition of high melting point nanoparticles to the aluminium alloy powders. Aluminium alloy powder particles for PBF-LB/M are typically in the 10–60 µm range. Throughout the whole paper, we will consequently differentiate between the terms “nanoparticles” for the additives and “particles” for the PBF-LB/M powders. Ball milling is one suitable method to add the nanoparticles to the aluminium powder. These nanoparticles act as solidification nuclei and suppress hot cracking. Table 1 gives an overview about several successful investigations regarding utilised aluminium alloys and nanoparticles. In some cases, the added nanoparticles act as nuclei themselves, and in other cases (Zr, Ti), the nanoparticles first react with the aluminium melt to form intermetallic compounds (Al_3_Zr, Al_3_Ti), which then act as nuclei.

In all these investigations, the nanoparticle types have been carefully chosen by suitable low crystal lattice mismatch between nuclei and aluminium matrix. However, the nanoparticles sizes, amounts and addition methods have been selected based on extensive experimental PBF-LB/M studies, requiring large amounts of modified powders (typically several kilograms). Our objective is to design promising powder modifications in small quantities (a few grams) before performing extensive PBF-LB/M studies. Therefore, we analyse the rapid melting/solidification behaviour of the modified powders in-situ by calorimetry. Especially differential fast scanning calorimetry (DFSC) with sample sizes of a few 10 µm particles (equivalent to PBF-LB/M powder particle sizes) and heating/cooling rates of up to 10^6^ K/s (equivalent to PBF-LB/M processes) is a suitable method [5]. In detail, rapid solidification conditions of single powder particles by DFSC differ from those in PBF-LB/M melt pools with dimensions in the several 100 µm ranges, where numerous powder particles are molten simultaneously. Nevertheless, we presume, that the main solidification behaviour can be approximated. Rapid solidification characteristics, e.g., undercooling, will then be correlated with grain sizes and crack characteristics in belonging PBF-LB/M components. This correlation shall be used for future purposeful powder material designs tailored for PBF-LB/M.

**Table 1:** Overview of several successful investigations regarding used aluminium alloys and nanoparticles.

Reference	Al Alloy	Nanoparticles
Gu et al., 2014 [6]	AlSi10Mg	TiC
Martin et al., 2017 [7]	7075	Zr → Al_3_Zr
Tan et al., 2020 [8]	2024	Ti → Al_3_Ti
Zhao et al., 2020 [9]	5024	TiC
Xi et al., 2020 [10]	AlSi10Mg	TiB_2_
Opprecht et al., 2020 [11]	6061	YSZ * (60 nm) → Al_3_Zr
Zhuravlev et al., 2021 [5]	7075	TiC (40 nm)
Heiland et al., 2021 [12]	7075	TiC (40 nm)
Mair et al., 2022 [13]	2024	CaB_6_ (200 nm)

* Yttrium stabilized zirconia Zr_(1−x)_Y_x_O_2._

## 2. Materials and Methods

**Figure 1:**
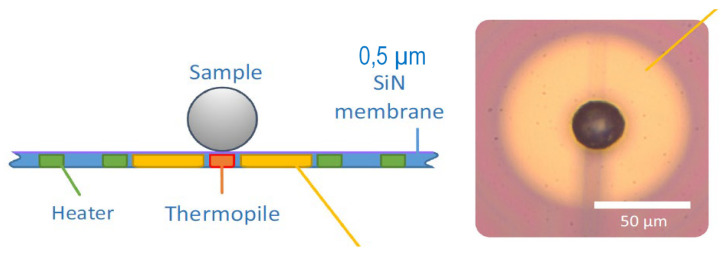
Schematic of the DFSC sensor (side view) as well as a light micrograph (top view) [5].

**Figure 2:**
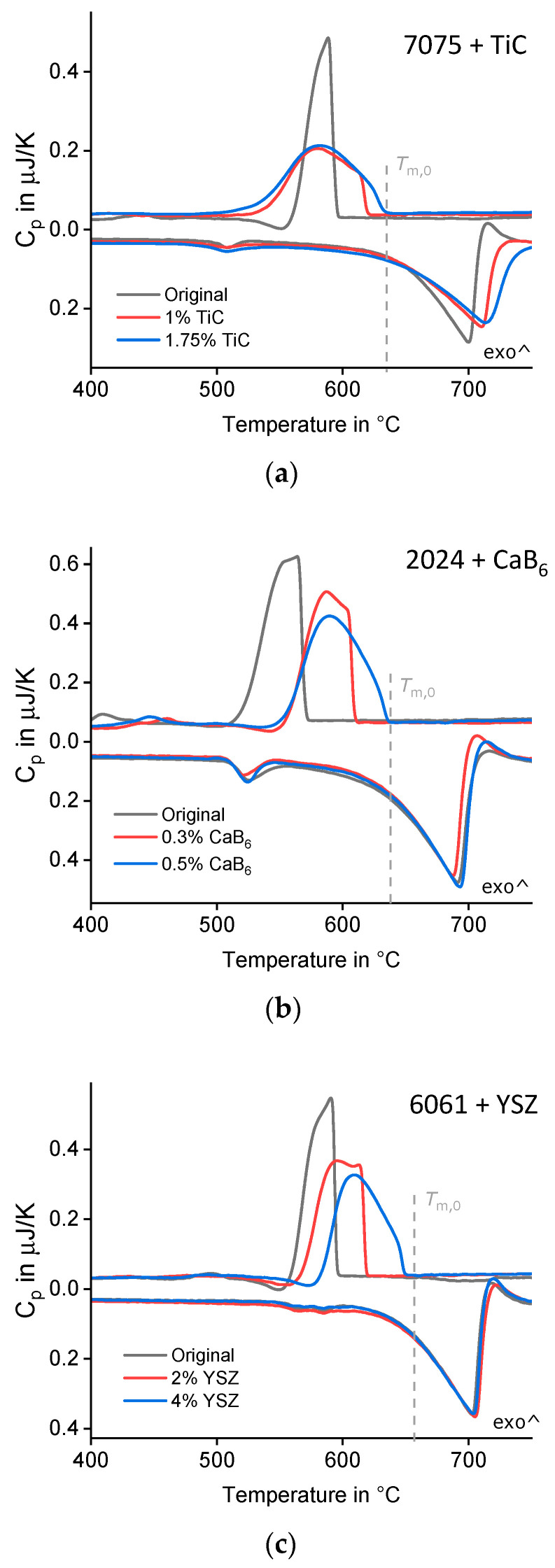
Exemplary DFSC heating and cooling curves (**a**) alloy 7075 without and with TiC nanoparticles, rate 10^4^ K/s. (**b**) Alloy 2024 without and with CaB_6_ nanoparticles, rate 10^4^ K/s. (**c**) alloy 6061 without and with YSZ nanoparticles, rate 10^3^ K/s. The vertical axis shows heat capacity in µJ/K, exothermal reactions upwards.

Table 2 contains the investigated aluminium alloys, nanoparticles and references. Nanoparticle amounts have been varied between zero and a few mass %. The rapid melting/solidification behaviour of several aluminium powders modified by different nanoparticles has been analysed in-situ by DFSC. Figure 1 shows a schematic DFSC sensor (side view) as well as a light microscope image (top view). The samples are individual powder particles with diameters of about 20 µm. Heating and cooling rates of 10^4^ K/s and maximum temperatures up to 823 °C have been used. DFSC measurements require careful temperature correction related to suitable reference temperatures. Details on measurement and evaluation have been published in [5]. Figure 2b shows three typical DFSC heating and cooling curves on alloy 2024 at a rate of 10^4^ K/s without and with CaB_6_ nanoparticles. The three bottom curves (endothermal) belong to melting. During rapid heating, we can see incipient melting slightly above 500 °C and a continuous ongoing melting up to melting finish at about 700 °C almost identical for all three variants. Melting finish depending on the heating rate has been extrapolated to heating rate zero, i.e., liquidus temperature [5] and is taken as reference temperature for undercooling (T_m,0_ = 638 °C dashed line). The three upper curves (exothermal) belong to solidification. Solidification of 2024 starts about 570 °C, i.e., undercooling amounts about 70 K, whereas solidification of 2024/0.3% CaB_6_ starts about 610 °C, i.e., undercooling amounts only about 30 K. In this example, the decrease of undercooling due to the nanoparticles is about 40 K. For the variant 2024/0.5% CaB_6_ rapid solidification starts almost without any undercooling. Figure 2a,c shows very similar behaviour in DFSC of single particles from alloys 7075/TiC and 6061/YSZ. Nanoparticle addition decreases solidification undercooling.

Whereas the rapid melting curves of 2024/CaB6 and 6061/YSZ (Figure 2b,c) are very similar, they differ for 7075/TiC (Figure 2a). This effect can arise from slightly different powder particle masses, from slightly different powder particle positions on the sensor and from different thermal contacts between powder particles and sensors. All DFSC curves have been temperature corrected accordingly [5], i.e., rapid solidification onset can be determined properly.

Each powder variant without and with nanoparticles has been analysed by DFSC on several individual powder particles. On each individual powder particle, several repeated heating/cooling cycles have been performed. Previous work has shown, that up to 300 repeated heating/cooling cycles can be performed on one individual aluminium powder particle without changing its melting/solidification behaviour [14]. Table 3 contains the number of analysed particles, number of repetitions per particle and the numbers of evaluated DFSC experiments for each variant. Typical undercooling scatter ranges from about ±20 K for high undercooling without nanoparticles to about ±2 K for low undercooling with nanoparticles.

Exactly the same nanoparticle-modified powder batches as in DFSC were used in PBF-LB/M processes. PBF-LB/M results and parameters are given in the references [5,11,12,13]. Additionally, the two powder variants 7021/TiC and 7021/TiB_2_ have been investigated with both methods in this work. Here, PBF-LB/M took place on an SLM 250^HL^ machine (SLM Solutions Group AG, Lübeck, Germany), equipped with a YLM-400-WC Laser (IPG Photonics, Oxford, MA, USA) by the following parameters: layer thickness 0.05 mm, hatch distance 0.08 mm, scan rate 900 mm/s, laser power 370 W, resulting in volume energy of 102.8 W/mm^3^. The specimens were fabricated under an argon atmosphere with a residual oxygen level of approximately 2000 ppm. Own data from [5,12] has also been re-evaluated regarding the above parameter set, i.e., all results on 7075 and 7021 shown below originate from identical PBF-LB/M parameters.

As-build samples have been analysed regarding grain size and crack characteristics by metallographic methods. The metallographic methods are also described in the references [5,11,12,13]. In these experiments, cracks have been described by different measures, i.e., crack density determined on cross sections by light microscopy [5,12], crack volume determined by X-ray microtomography [13] and total crack length per area determined on cross sections by light microscopy [11]. To compare these different crack measures, we suggest a crack characteristic value C, which is defined by the ratio of the crack measure with nanoparticles to the maximum crack measure without nanoparticles, equations (1–3). In further evaluation, we propose that these crack characteristic values C_D_, C_V_ and C_L_ can be directly compared, i.e., we call them just crack characteristic value C. By this definition the crack characteristic value C can exist in the range of 0 to 1, with C = 0 meaning complete crack suppression by nanoparticles and C = 1 meaning no change in cracking with nanoparticles. This relative crack characteristic C can be used to compare different crack measures, different aluminium alloys, different nanoparticles and different PBF-LB/M processes.

(1) $$C_{D}=\frac{{\left(crack\;density\right)}_{with\;NP}}{{\left(crack\;density\right)}_{without\;NP}}$$

for 7075/TiC, 7021/TiC, 7021/TiB_2_ data from [5,12] and this work

(2) $$C_{V}=\frac{{\left(crack\;volume\right)}_{with\;NP}}{{\left(crack\;volume\right)}_{without\;NP}}$$

for 2024/CaB_6_, data from [13].

(3) $$C_{L}=\frac{{\left(total\;crack\;length\right)}_{with\;NP}}{{\left(total\;crack\;length\right)}_{without\;NP}}$$

for 6061/YSZ, data from [11].

Single rapidly solidified particles from DFSC were analysed by SEM and TEM. The metallographic preparation route for SEM analysis of such small and individual powder particles has been developed as described in [15]. For TEM investigations of solidified particles, the particles were first embedded in epoxy, then a FEI Helios G4 focused ion beam (FIB) instrument was used to cut a thin section through the particle using the standard lift-out approach. A double aberration corrected JEOL ARM-200F was used for scanning transmission electron microscopy (STEM) and elemental mapping of the particle surface with electron energy loss spectroscopy (EELS).

## 3. Results and Discussion

DFSC solidification undercooling and PBF-LB/M crack characteristics C, as well as grain size have been investigated for each individual variant of aluminium alloy, nanoparticle type and nanoparticle amount according to Table 2. All powder variants will be compared in terms of crack characteristics C, grain size and solidification undercooling.

### 3.1. Correlation between Crack Characteristics C and Solidification Undercooling

**Figure 3:**
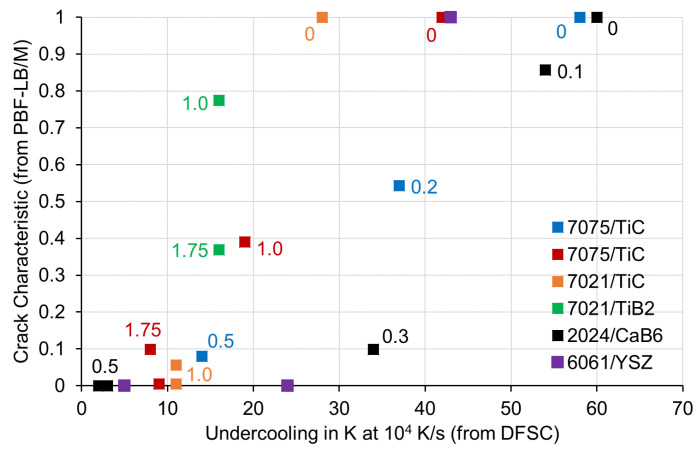
Correlation between crack characteristics C and solidification undercooling. Data points are labelled with the belonging nanoparticle amounts in mass %. Relative crack characteristics of 2024/CaB_6_ and 6061/YSZ have been calculated from [11,13].

Figure 3 shows the correlation between crack characteristics C and solidification undercooling. Each individual point displays crack characteristics and solidification undercooling for one powder variant. Results from the same aluminium alloy/nanoparticle system with different nanoparticle amounts are plotted in the same colour. Data points are labelled with the belonging nanoparticle amounts in mass %. In most cases, undercooling, as well as crack characteristics decrease with increasing nanoparticle amount. The only exception is the powder variant 7021/TiB_2_, which even at a high amount of 1.75 mass % reduced undercooling only to 16 K and crack characteristic value only to about 0.4.

**Figure 4:**
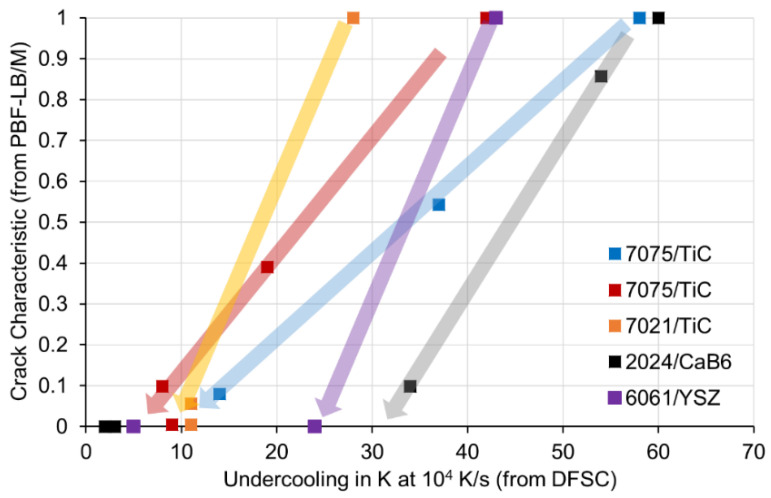
Correlation between crack characteristics C and solidification undercooling with arrows as guides for the eye. Relative crack characteristics of 2024/CaB_6_ and 6061/YSZ have been calculated from [11,13].

In Figure 4, we have added arrows as a guide for the eye, each arrow belonging to one aluminium alloy/nanoparticle system. Now it can be clearly seen, that crack characteristic C decreases with decreasing undercooling for all variants. For a given aluminium alloy/nanoparticle system, undercooling decreases with an increasing number of nanoparticles. The effect of undercooling on crack characteristics depends on the individual aluminium alloy/nanoparticle system, which can be explained by the different PBF-LB/M machines and parameters as well as crack measures used. In the bottom left corner of the diagram, we find a successful process window relative consistent for all investigated variants. A low solidification undercooling of less than about 10–15 K in DFSC (at a cooling rate of 10^4^ K/s) correlates with almost crack-free PBF-LB/M components. This correlation can be used in future for the purposeful design of powder materials in small quantities (a few grams) before conducting extensive PBF-LB/M studies.

### 3.2. Correlation between Grain Size and Solidification Undercooling

**Figure 5:**
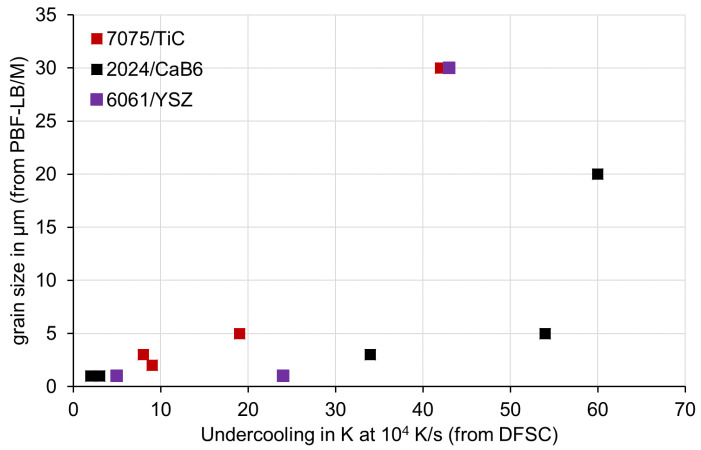
Correlation between grain size and solidification undercooling. Grain sizes of 2024/CaB_6_ and 6061/YSZ have been adopted from [11,13].

Figure 5 shows the correlation between grain size and solidification undercooling. Each individual point displays grain size and solidification undercooling for one powder variant. Please have in mind, that grain sizes result from PBF-LB/M samples and therefore can be larger than the individual powder particles in DFSC. Results from the same aluminium alloy/nanoparticle system with different nanoparticle amounts are plotted in the same colour. Like crack characteristic C, grain size decreases with decreasing undercooling, proving that the nanoparticles are responsible for efficient inoculation. Besides decreasing grain size, also grain geometry changes from columnar to equiaxed with the increasing number of nanoparticles [5,11,12,13]. In the case of columnar grains, the column widths have been plotted in Figure 5. Column lengths have grown even larger in the range of several 100 µm. In the bottom left corner of the diagram, we find again a successful process window relative consistent for all investigated variants. A low solidification undercooling of less than about 10–15 K in DFSC (at a cooling rate of 10^4^ K/s) correlates with low grain sizes of less than about 3 µm.

### 3.3. Correlation between Crack Characteristics C and Grain Size

**Figure 6:**
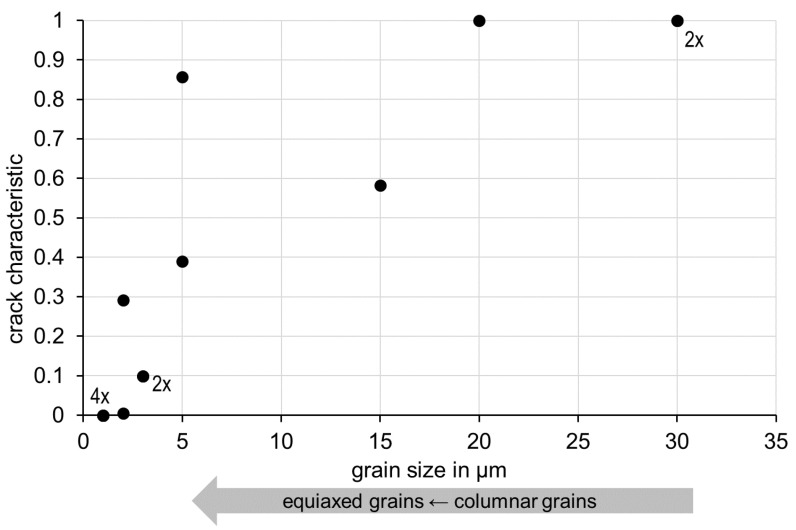
Correlation between crack characteristics C and grain size. Arrow indicates concurrent change from large columnar to small equiaxed grains. Some data points overlap in this diagram and have been marked accordingly (2×, 4×).

Finally, we have correlated crack characteristics C and grain size independent of the alloy/nanoparticle system (Figure 6). Some data points crack characteristic/grain size overlap in this diagram and have been marked accordingly (2×, 4×). As expected, crack characteristic C decreases with decreasing grain size. Moreover, in this diagram we find a successful process window in the bottom left corner which is relatively consistent for all investigated variants. Low grain size of less than about 3 µm correlates with almost crack-free PBF-LB/M components. As mentioned above, grain shape changes concurrently with grain size from large columnar grains to small equiaxed grains. 

This correlation is in good agreement with the accepted mechanism of hot cracking during solidification [2,3,4]. Large columnar dendrites form a solid network early during solidification, which encloses the remaining encapsulated melt volumes. During further solidification of these encapsulated melt volumes, their shrinkage cannot be sufficiently accommodated by the deformation of the solid network and hot cracking occurs in these places. Small equiaxed dendrites form such a solid network significantly later during solidification. The remaining melt volumes are not encapsulated early. Instead, they are interconnected and can balance volume shrinkage. This correlation confirms our presented approach, to use DFSC on small quantities (a few grams) for future purposeful design of powder materials for crack-free PBF-LB/M components.

### 3.4. Efficiency of Nanoparticle Inoculation

**Figure 7:**
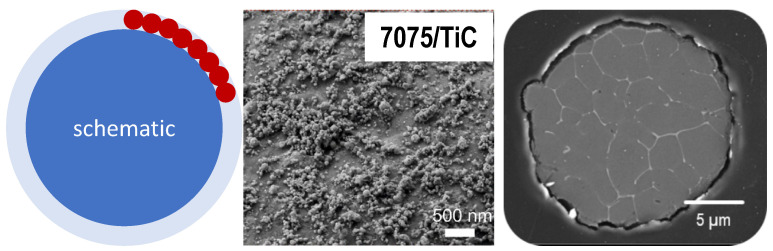
(**Left**) Schematic of a monolayer of nanoparticles. (**Middle**) Surface of a powder particle 7075/TiC. (**Right**) Cross section of an individual powder particle 7075/TiC, rapidly solidified by DFSC.

Further, we make an estimation of the inoculation efficiency of nanoparticles on powder particle surfaces during PBF-LB/M. Table 4 gives the necessary amounts of nanoparticles for successful PBF-LB/M to suppress hot cracks in the present work, as well as from [10,12]. In many cases, about a few mass % of nanoparticles are required. The following calculation illustrates the consequences. Let us assume a typical aluminium particle diameter for PBF-LB/M of *d_p_* = 40 µm and a typical nanoparticle dimension of *d_np_* = 40 nm (Table 2). Let us further assume a continuous monolayer of nanoparticles on the aluminium powder particle surface (Figure 7 left). In this case, the content of nanoparticles c_np_ in mass % can be approximated by Equation (4) considering the particle density *ρ*_p_ = 2.7 g/cm^3^ (aluminium) and the nanoparticle density *ρ_np_* = 4.9 g/cm^3^ (TiC).

(4) $$c_{np}=\frac{\rho_{np}}{\rho_{p}}\cdot \frac{{\left(d_{p}+2\cdot d_{np}\right)}^{3}-d_{p}^{3}}{d_{p}^{3}}$$

Under the mentioned assumptions, one monolayer of TiC nanoparticles on aluminium alloy 7075 particles equals an amount of about 0.6 volume%, corresponding to about 1.0 mass % nanoparticles. Figure 7 (middle) shows an SEM image of the surface of a 7075/TiC powder particle with 1 mass % TiC. The powder particle surface is almost completely covered by nanoparticles, which proves that the above approximation is realistic. We can see some gaps, but in other areas, the nanoparticles build multilayers. Next, we estimate the number of nanoparticles *N_np_* in the monolayer according to Equation (5).

(5) $$N_{np}=\frac{{\left(d_{p}+2\cdot d_{np}\right)}^{3}-d_{p}^{3}}{d_{np}^{3}}$$

This estimation results in about 10^6^ nanoparticles per aluminium powder particle, i.e., about 10^6^ potential nuclei for solidification. For comparison, Figure 7 (right) shows a SEM image of a metallographic cross section of one individual powder particle of 7075/TiC with a diameter of about 20 µm, solidified by DFSC with a cooling rate of 10^4^ K/s. The dendrite arm size respectively grain size amounts to a few µm and corresponds very well to the grain size in crack-free PBF-LB/M components (Figure 6). This is another indication, that rapid single-particle solidification by DFSC resembles melt pool conditions in PBF-LB/M. In Figure 7 right, we can only very roughly guess the number of efficient solidification nuclei, especially because we see a certain number of dendrite arms, but we do not know their crystal orientation. However, even if we very generously guess the number of active nuclei to be in the order of 10 to 100, we can conclude on a very low nucleation efficiency of about 10^−5^ to 10^−4^ for such nanoparticles added by ball milling.

**Figure 8:**
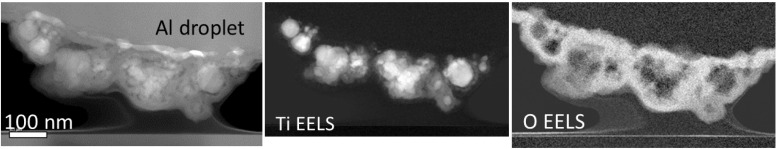
STEM/EELS cross section image, which has been prepared by FIB from a single 7075/TiC particle after rapid solidification by DFSC.

The reason for this low nucleation efficiency is unclear at the moment. One assumption is, that the nanoparticles added by ball milling stick to the relative stable natural oxide layer of the aluminium powder particles, which may hinder direct contact with the aluminium melt. This assumption is supported by the STEM/EELS cross-section image in Figure 8, which has been prepared from a single 7075/TiC particle after rapid solidification by DFSC. We can clearly see the TiC nanoparticles with dimensions of about 40 nm as well as the oxide layer in between. This assumption needs further investigation, but in case it is realistic, it would require other, more effective inoculation methods, i.e., adding nanoparticles inside the powder particle volumes [16]. This could drastically reduce the number of nanoparticles required to achieve a reduced solidification undercooling.

## 4. Conclusions

Different high-strength aluminium alloy powders modified with different nanoparticles by ball milling (7075/TiC, 2024/CaB_6_, 6061/YSZ) have been investigated in-situ during rapid solidification by DFSC. Solidification undercooling has been evaluated and was found to decrease with increasing number of nanoparticles. Solidification undercooling from DFSC was compared with PBF-LB/M results from the same powder batches regarding hot cracking and grain size. Low solidification undercooling correlates with little hot cracking and small grains. Quantitatively, solidification undercooling less than about 10–15 K correlates with almost crack free PBF-LB/M components and grain sizes less than about 3 µm. This correlation will be used for future purposeful powder material design on small quantities before performing extensive PBF-LB/M studies.

Nanoparticles added on powder particle surfaces by ball milling exhibit a very low nucleation efficiency during rapid solidification in the range of 10^−5^ to 10^−4^ (active nuclei related to total number of nanoparticles). The reason for this low nucleation efficiency shall be further investigated and other inoculation methods instead of ball milling shall be considered for higher nucleation efficiency such that the amount of added nanoparticles can be greatly reduced while retaining the improved properties of PBF-LB/M components.

## Figures and Tables

**Table 2:** Investigated aluminium alloys, nanoparticles and references.

Reference	Al-Alloy	Nanoparticles, Amount	NP Deposition	DFSC Rate
Zhuravlev et al., 2021 [5]	7075	TiC 40nm 0–0.5 mass %	ball milling wet deposition	10^4^ K/s
Heiland et al., 2021 [12]	7075	TiC 40 nm 0–2.5 mass %	ball milling	10^4^ K/s
this work	7021	TiC 40 nm 0–1.75 mass %	ball milling	10^4^ K/s
this work	7021	TiB_2_ 50 nm 0–1.75 mass %	ball milling	10^4^ K/s
Mair et al., 2022 [13]	2024	CaB_6_ 200 nm 0–2 mass %	ball milling	10^4^ K/s
Opprecht et al., 2020 [11]	6061	YSZ 60 nm 0–4 volume % *	ball milling	10^3^ K/s

* Density of YSZ is about 6 g/cm^3^, i.e., roughly double the density of aluminium.

**Table 3:** Numbers of analysed particles, numbers of repetitions per particle and numbers of evaluated DFSC experiments for each variant.

Alloy/Nanoparticles	Numbers of Analysed Particles	Numbers of Repetitions per Particle	Numbers of Evaluated DFSC Experiments
7075/TiC	at least 5	about 60	about 300
2024/CaB_6_	at least 3	about 75	about 225
6061/YSZ	at least 15	about 10	about 150

**Table 4:** The necessary content of nanoparticles for successful PBF-LB/M to suppress hot cracks.

Reference	Alloy/Nanoparticles	Necessary Amount of Nanoparticles
this work	7075/TiC	1.75 mass %
this work	7021/TiC	1 mass %
Mair et al., 2022 [13]	2024/CaB_6_	0.5 mass %
Opprecht et al., 2020 [11]	6061/YSZ	2 volume % *

* Density of YSZ is about 6 g/cm^3^, i.e., roughly double the density of aluminium.

## References

[1] Dimitrii A., Effenberg G. (1993). Petrov and MSIT®. SpringerMaterials.

[2] Kurz W., Fisher D.J. (1998). Fundamentals of Solidification.

[3] Coniglio N., Cross C.E. (2013). Initiation and growth mechanisms for weld solidification cracking. Int. Mater. Rev..

[4] Rappaz M., Drezet J., Gremaud M. (1999). A new hot-tearing criterion. Metall. Mater. Trans. A.

[5] Zhuravlev E., Milkereit B., Yang B., Heiland S., Vieth P., Voigt M., Schaper M., Grundmeier G., Schick C., Kessler O. (2021). Assessment of AlZnMgCu alloy powder modification for crack-free laser powder bed fusion by differential fast scanning calorimetry. Mater. Des..

[6] Gu D., Wang H., Chang F., Dai D., Yuan P., Hagedorn Y.-C., Meiners W. (2014). Selective laser melting additive manufacturing of TiC/AlSi10Mg bulk-form nanocomposites with tailored microstructures and properties. Phys. Procedia.

[7] Martin J.H., Yahata B.D., Hundley J.M., Mayer J.A., Schaedler T.A., Pollock T.M. (2017). 3D printing of high-strength aluminium alloys. Nature.

[8] Tan Q., Zhang J., Sun Q., Fan Z., Li G., Yin Y., Liu Y., Zhang M.-X. (2020). Inoculation treatment of an additively manufactured 2024 aluminium alloy with titanium nanoparticles. Acta Mater..

[9] Zhao T., Dahmen M., Cai W., Alkhayat M., Schaible J., Albus P., Zhong C., Hong C., Biermann T., Zhang H. (2020). Laser metal deposition for additive manufacturing of AA5024 and nanoparticulate TiC modified AA5024 alloy composites prepared with balling milling process. Opt. Laser Technol..

[10] Xi L., Gu D., Guo S., Wang R., Ding K., Prashanth K.G. (2020). Grain refinement in laser manufactured Al-based composites with TiB2 ceramic. J. Mater. Res. Technol..

[11] Opprecht M., Garandet J.-P., Roux G., Flament C., Soulier M. (2020). A solution to the hot cracking problem for aluminium alloys manufactured by laser beam melting. Acta Mater..

[12] Heiland S., Milkereit B., Hoyer K.-P., Zhuravlev E., Kessler O., Schaper M. (2021). Requirements for successfully processing high-strength AlZnMgCu alloys with PBF-LB/M. Materials.

[13] Mair P., Kaserer L., Braun J., Stajkovic J., Klein C., Schimbäck D., Perfler L., Zhuravlev E., Kessler O., Leichtfried G. (2022). Dependence of mechanical properties and microstructure on solidification onset temperature for varying Al2024/CaB_6_ mixtures processed using laser powder bed fusion. Mater. Sci. Eng. A.

[14] Peng Q., Yang B., Milkereit B., Liu D., Springer A., Rettenmayr M., Schick C., Keßler O. (2021). Nucleation Behavior of a Single Al-20Si Particle Rapidly Solidified in a Fast Scanning Calorimeter. Materials.

[15] Milkereit B., Meißner Y., Ladewig C., Osten J., Peng Q., Yang B., Springer A., Keßler O. (2021). Metallographic Preparation of Single Powder Particles. Pract. Metallogr..

[16] Hengsbach F., Hoyer K.-P., Schaper M., Andreiev A. (2021). Isotropic, Crack-Free Steel Design Using an Additive Manufacturing Method.

